# Annotations

**Published:** 1908-05-23

**Authors:** 


### ANNOTATIONS.

### Cosmopolitan Quackery.

It is a pity that the report of the Australian Royal Commission on Secret Drugs, Cures and Foods is not more widely known in this country. Medical men who are interested in this important social problem will find the Report in various medical libraries, such, for instance, as the reading room of the Royal College of Physicians. Quackery is there exposed in all its forms, and the vast extent of the evil is clearly demonstrated. The editor of *Truth*, dealing with the cosmopolitan nature of quackery, suggests that this report should be republished in England by the Home Office, in the form of a Government Blue Book; since the need for legislation is just as great here as in Australia, and "all the biggest rogues in this pillory are at work among us every day." The article shows up a particularly blatant German nostrum, which has recently been flooding the press in this country as an alleged specific for neurasthenia, and illustrates, by means of this and kindred remedies, that cosmopolitanism which is the hallmark of up-to-date quackery. A series of articles now appearing in The Hospital has already drawn attention to this "dumping" of foreign secret remedies upon our shores. Active legislation is imperatively needed because the evil is increasing from year to year. But legal measures in this matter are, we fear, likely to be deferred from Parliament to Parliament, so long as public opinion, as expressed by the words of some of our judges and the actions of our principal newspapers, remains as tolerant as it is at present of the quack and all his works. It is to be regretted that there is a section of the educated public which believes that much of the medical profession's condemnation of quackery is inspired by sordid motives of professional jealousy. Unworthy suspicions of this sort are hard to deal with, because they can seldom be met and defeated in fair fight.

### The Royal Society and Cancer Research.

The scientific exhibits at the annual conversazione given by the Royal Society on May 13 at Burlington House were of more than usual interest. The collection illustrating the experimental study of cancer, shown by Dr. E. F. Bashford on behalf of the authorities of the Imperial Cancer Research Fund attracted great attention. This publicity given by the Royal Society to the investigation of cancer will undoubtedly do good in various directions. In the first place the public will thereby learn indirectly of the good work that is being done by the Cancer Research Fund, and of the extent and prestige of that organisation. In the second place, this exhibit, under the auspices of the Royal Society, will draw increased attention to the case for the experimental study of disease in animals. A series of microphotographs, pathological specimens and drawings showed clearly that cancer in man and cancer in animals run parallel courses, and that the disease is comparable in the two cases. All the characteristic lesions of cancer in man can be reproduced in the mouse. This demonstration of the practical identity of cancer in different members of the animal kingdom is of great significance.

### A Remarkable Career.

Dr. Theodore Duka, who died recently at Bournemouth in his eighty-third year, had a very remarkable personality. He was by birth a Hungarian of noble family, and, we learn from the *Times* that in early life he played an active and almost romantic part in the political and military vicissitudes of his native country in 1848 and 1849. He was personal aide-de-camp to General Görgey, and at the battle of Komoru he was decorated on the field with the Order of Valour for a conspicuous act of courage. When the Hungarian forces finally surrendered at Villágos Dr. Duka became a prisoner of war; but he eventually escaped, and, after a long series of adventures and wanderings in Austria and Germany, proceeded to London in 1850. Here he adopted the profession of medicine, and for the rest of his career identified himself with this country and became a British subject in 1854. He studied at St. George's Hospital, and in 1853 he took the degrees of M.R.C.S. (Eng.) and M.D. (St. Andrews). In 1854 he obtained a commission on the medical staff of the Bengal Army, and served throughout the Mutiny. He retired from the service in 1877 with the military rank of lieutenant-colonel. Dr. Duka finally settled in London and became an active member of various learned societies. In 1883 the Emperor Francis Joseph conferred upon him the Order of the Iron Crown.

### The Infant's Binder.

Dr. A. Waring, of Nottingham, recently read a paper upon "The Dangers and Evil Effects of Infant Binders." The author condemns the ancient practice of enveloping the infant body in "countless bands, swades, and swaddling clothes." He maintains that warmth can be as well supplied by loose clothing, while support is not needed, and the ligatured umbilical cord is better protected during the first weeks of life by some antiseptic dusting powder. The binder of every kind is, in Dr. Waring's opinion, not only unnecessary, but in many cases positively harmful. He asserts that chronic discomfort, vomiting, inguinal and umbilical hernia, hæmorrhoidal distension of veins, and prolapsus ani, convulsions, and arrest of growth and development of the chest are all frequently caused by the constriction and compression of binders. Furthermore, he believes that the dangers of overlaying are increased by the use of tight thoracic binders. He considers that binder compression, which throws the whole pressure of the abdominal contents upon the inguinal region, is a far commoner cause of inguinal hernia than is phimosis. Dr. Waring's indictment of the binder, although interesting and suggestive, is not much more than a series of allegations. He does not bring forward any convincing evidence in support of them. It seems reasonable to suppose that tight binders occasionally have some share in the production of the graver evils which he names; but we hesitate to believe that these evils are other than exceptional results of the application of the binder.

